# Multiple Statistical Analysis Techniques Corroborate Intratumor Heterogeneity in Imaging Mass Spectrometry Datasets of Myxofibrosarcoma

**DOI:** 10.1371/journal.pone.0024913

**Published:** 2011-09-29

**Authors:** Emrys A. Jones, Alexandra van Remoortere, René J. M. van Zeijl, Pancras C. W. Hogendoorn, Judith V. M. G. Bovée, André M. Deelder, Liam A. McDonnell

**Affiliations:** 1 Biomolecular Mass Spectrometry Unit, Department of Parasitology, Leiden University Medical Center, Leiden, The Netherlands; 2 Department of Pathology, Leiden University Medical Center, Leiden, The Netherlands; Consejo Superior de Investigaciones Cientificas, Spain

## Abstract

MALDI mass spectrometry can generate profiles that contain hundreds of biomolecular ions directly from tissue. Spatially-correlated analysis, MALDI imaging MS, can simultaneously reveal how each of these biomolecular ions varies in clinical tissue samples. The use of statistical data analysis tools to identify regions containing correlated mass spectrometry profiles is referred to as imaging MS-based molecular histology because of its ability to annotate tissues solely on the basis of the imaging MS data. Several reports have indicated that imaging MS-based molecular histology may be able to complement established histological and histochemical techniques by distinguishing between pathologies with overlapping/identical morphologies and revealing biomolecular intratumor heterogeneity. A data analysis pipeline that identifies regions of imaging MS datasets with correlated mass spectrometry profiles could lead to the development of novel methods for improved diagnosis (differentiating subgroups within distinct histological groups) and annotating the spatio-chemical makeup of tumors. Here it is demonstrated that highlighting the regions within imaging MS datasets whose mass spectrometry profiles were found to be correlated by five independent multivariate methods provides a consistently accurate summary of the spatio-chemical heterogeneity. The corroboration provided by using multiple multivariate methods, efficiently applied in an automated routine, provides assurance that the identified regions are indeed characterized by distinct mass spectrometry profiles, a crucial requirement for its development as a complementary histological tool. When simultaneously applied to imaging MS datasets from multiple patient samples of intermediate-grade myxofibrosarcoma, a heterogeneous soft tissue sarcoma, nodules with mass spectrometry profiles found to be distinct by five different multivariate methods were detected within morphologically identical regions of all patient tissue samples. To aid the further development of imaging MS based molecular histology as a complementary histological tool the Matlab code of the agreement analysis, instructions and a reduced dataset are included as supporting information.

## Introduction

**Figure 1:**
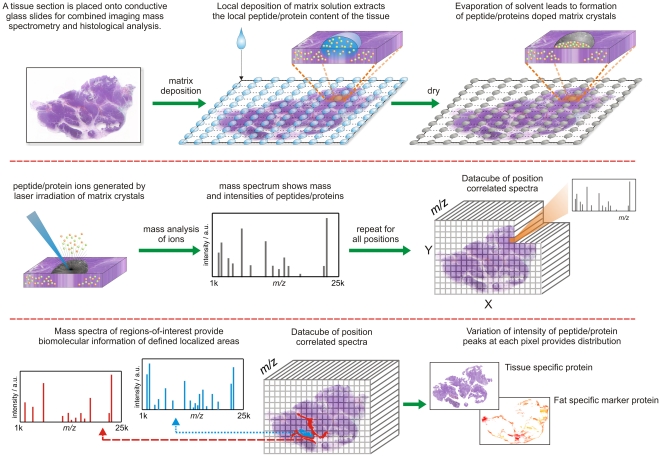
Schematic of a MALDI Imaging MS experiment.

MALDI mass spectrometry of tissue sections can generate profiles that contain hundreds of distinct biomolecular ions [1], [2]. The tissue section is prepared for MALDI analysis by the deposition a matrix solution, which seeps into the tissue dissolving an assortment of biomolecules (peptides, proteins, metabolites, lipids). As the solvent evaporates the dissolved biomolecules are extracted from the tissue, Figure 1. Further evaporation leads to crystallization of the matrix and the formation of biomolecule-doped matrix crystals. Irradiation of the matrix crystals with a pulsed ultra-violet laser leads to efficient production of gas phase biomolecular ions. Mass spectrometry separates these ions according to their mass, thus providing the ability to distinguish between biomolecules of different mass and to simultaneously measure their mass.

MALDI-MS of a localized position on a tissue section generates a mass spectrum containing many of the biomolecules present at that position [3]. The mass spectra of an array of positions across the tissue section describe the spatial variation of every biomolecular ion detected from the tissue [4], [5], Figure 1. Such spatially resolved analysis is referred to here as imaging MS. The dataset of position-correlated mass spectra can be aligned with an optical image of the histologically stained tissue [6], [7] to allow the distributions of specific biomolecular ions to be compared with the tissue section's morphology, or the biomolecular ions detected from specific pathohistological entities to be interrogated for the identification of new candidate biomarkers [8]. Using essentially the same technique but different sample (tissue) preparation protocols imaging MS can be used to analyze peptides, proteins, lipids and metabolites [9].

Ionization biases are prevalent in mass spectrometry analysis of complex mixtures [10]; peptide (and protein) purification and separation technologies are routinely used to increase the number of species detected in a mass spectrometry experiment [11]. Such liquid based separation strategies are of limited utility for imaging MS because of the need to retain spatial integrity and the extremely small amounts of tissue analyzed in each pixel: a single 100×100 µm pixel contains just 25 average sized, 20 µm, cells. The ability of imaging MS to detect hundreds of peptides and proteins directly from a tissue section is testament to the successful on-tissue fractionation that occurs during sample preparation. Nevertheless, even if hundreds of distinct species can be detected significant ionization biases can remain. Stoeckli *et al.* have demonstrated that if the relative response factors of an analyte in different tissues can be determined then imaging MS can provide relative quantification [12]. These experiments concerned the analysis of pharmaceuticals in whole body tissue sections; the relative response factors were determined by homogeneously depositing the pharmaceutical on to whole-body sections from an undosed animal. MALDI imaging MS of the uniformly coated whole body tissue section did not generate a uniform MALDI signal of the pharmaceutical. When the relative response factors were calculated from the relative signal deviations, and then applied to MALDI imaging MS results obtained from a dosed animal, the relative quantitation was consistent with results obtained using whole body autoradiography.

The simultaneous determination of relative response factors for all peptides and proteins detected from tissue is much more challenging (and to these authors' knowledge has not been performed to date); it would require isotopically labelled analogues of all detected peptides and proteins to be added as internal standards as well as a significant increase in the peak capacity of the mass spectrum to resolve every component. Owing to the lack of practical quantitation strategies peptide and protein imaging MS experiments typically compare the MS signals (after a number of preprocessing and normalization steps [13], [14]). Reproducible sample preparation is central to this approach and a number of automated sample preparation stations have been developed to provide the necessary capabilities [5]. Multiple studies have now demonstrated how imaging MS combined with histopathological annotation can be used to identify new candidate biomarkers [8], [15], [16], [17]. Note: potential ionization biases within a heterogeneous tissue means that it is vital to independently validate any biomarkers found to be associated with specific histopathological entities, to ensure that the differential signals are not due to the different chemical background of the histopathological entity.

The ability of imaging MS to detect hundreds of peptides and proteins, and the sensitivity of their signals to the underlying biomolecular content of the tissue, provides new opportunities for annotating clinical tissues. There is growing awareness that imaging MS can be used to annotate tissues based solely on the detected MS profiles and thereby differentiate regions that are not distinct using established histopathological tools but which are characterized by different MS signatures [18], [19], [20]. Such capabilities have several important clinical applications:

- Identification of sub-regions within tumors (intratumor heterogeneity) [18], [20].
- Differentiation between tumors with overlapping morphology (i.e. distinct disease entities) [17], [20], [21].
- Characterization of tumor-interface zones (regions of greatest vascularization and most active growth) [19], [22], [23].

Deininger *et al.* were among the first to explicitly describe this potential of imaging MS to complement established histopathological methods [18]. A hierarchical cluster analysis of an imaging MS dataset of intestinal type gastric cancer revealed a detailed clustering that was postulated as arising from the tumor's subclones. The identification of regions of tissue that are characterized by distinct mass spectrometry profiles is now referred to as molecular histology. Formally, histology refers to the study of the microscopic anatomy of tissue. For example a histopathological examination of soft tissue sarcomas uses, amongst others, cellular phenotype, pleomorphism, and cellularity for tumor classification and differentiation, mitotic rate, and necrosis to grade the tumor [24], [25]. The spatial resolution currently used in most imaging MS experiments, pixel size ≥50 µm to maintain high sensitivity for peptide and protein mass spectrometry [5], [26], is insufficient to resolve all microscopic features. Higher spatial resolution analyses have already been reported for tissues containing abundant peptides and proteins [27], [28]. As the field develops further the sensitivity will improve enabling imaging MS to routinely resolve more of the microscopic features utilized in current histological practice.

A recent imaging MS-based molecular histology analysis of myxofibrosarcoma revealed intratumor heterogeneity in the imaging MS datasets from multiple patients [20] that was consistent with the multistep genetic progression clonal development hypothesis for this sarcoma [29]. Hierarchical cluster analysis of an imaging MS dataset comprising low-grade, intermediate-grade and high-grade myxofibrosarcoma revealed that the intermediate-grade tumor contained discrete nodules whose MS profiles resembled high-/low-grade myxofibrosarcoma. A support-vector machine classifier, created using six localized regions within a single imaging MS dataset of intermediate-grade myxofibrosarcoma, was then applied to datasets from additional intermediate-grade patient tissue samples. A nodular structure was revealed within each dataset, and which further subdivided the regions indicated as high-grade-like and low-grade-like by hierarchical cluster analysis. The intratumor heterogeneity in the imaging MS datasets of intermediate grade myxofibrosarcoma provides further evidence that imaging MS may complement established histological and histochemical methods by revealing previously unknown biomolecular variation.

The hundreds of peptides and proteins detected by imaging MS provide new opportunities for annotating tissues based on their MS profiles but also new challenges. Data analysis methods are required that reveal distinct regions within the imaging MS datasets. A number of techniques have been investigated, including the multivariate techniques principal component analysis (PCA) [30], independent component analysis (ICA) [31], co-localization analysis [13], non-negative matrix factorization (NNMF), probabilistic latent semantic analysis (PLSA) [31] and the clustering techniques k-means [32] and hierarchical clustering [18]. This array of algorithms provides the user with a veritable data-analysis-toolbox with which to analyze imaging MS data but also raises uncertainty. The data analysis methods have different assumptions about the nature of the data (e.g. PCA assumes normally distributed data whereas ICA does not), optimize different functions and are based on different algorithms. Consequently their results can differ in both nature (which regions of the imaging MS dataset are distinct) and in order (which output contains a specific region found to be distinct) [30], [31], [33]. This dependence on the data analysis technique raises questions about the reliability of an analysis based on any single method. For example, are the regions of an imaging MS dataset highlighted by the third output of a PCA analysis, but not by PLSA, truly distinct? Such uncertainty has left imaging MS-based molecular histology in the testing stage of its development, and so most reports have focused on tissues containing well differentiated morphologies that allow histological verification of the regions identified by the analysis [31], [32]. For imaging MS-based molecular histology to complement established histological practice data analysis tools are required that provide additional discriminative capabilities.

We postulated that those regions of a tissue's imaging MS dataset found to be distinct by several multivariate methods could provide a more robust data analysis strategy for imaging MS-based molecular histology, by preferentially highlighting those regions consistently identified as having distinct MS profiles. Here it is demonstrated how data reduction by automated feature detection enables an array of multivariate techniques to be applied and compared. It is then shown how the regions of an imaging MS dataset consistently identified by five multivariate methods as having distinct MS profiles provides a consistently accurate summary of the heterogeneity. The application of this agreement analysis to imaging MS datasets from multiple intermediate grade myxofibrosarcoma patient tissue samples reveals distinct nodules in morphologically identical tissue.

## Methods

### Tissue/clinicopathological data

Slides were re-evaluated histologically and classified according to the 2002 World Health Organization criteria [25], then graded according to the French Fédération Nationale des Centres de Lutte Contre le Cancer (P.C.W.H) [24]. All tissue samples were handled in a coded fashion and were no longer required for patient diagnosis. Following Dutch national ethical guidelines (Code for proper secondary use of human tissue, Dutch Federation of Medical Scientific Societies, http://www.federa.org/fmwv-english) explicit ethical and informed consent are not required for such excess, anonymized tissues.

### Tissue preparation

Tumor tissue samples obtained from surgical resection specimens were snap frozen in liquid isopentane and then stored at −80°C until sectioning. 5 µm thick tissue sections were cut at −20°C using a cryomicrotome and stained with hematoxylin & eosin (H&E) to check diagnosis and viability of the tissue.

For the MALDI imaging MS experiments 12 µm thick tissue sections were cut at −20°C and thaw mounted onto conductive glass slides (Delta Technologies, Stillwater, USA). The tissues were then slowly brought to room temperature in a desiccator and prepared for MALDI analysis of the tissue's peptides and proteins. The tissues were washed in isopropanol and sinapinic acid (SA) matrix was added using an ImagePrep (Bruker Daltonics, Bremen, Germany) and a 20 mg/ml solution of sinapinic acid in 6∶4 AcN∶0.5% TFA (*aq.*). A detailed outline of the ImagePrep settings used for matrix deposition is supplied as supporting information (see file Supporting Information S1).

### Mass spectrometry

All peptide and protein imaging MS experiments were performed using an Autoflex III mass spectrometer (Bruker Daltonics, Bremen, Germany) and were acquired in fully automated mode using the Flex software suite (FlexControl 3.0, FlexImaging 2.1, FlexAnalysis 3.0, Bruker Daltonics). The experiments were performed in positive-ion, linear mass analyzer mode using 100 µm pixel size, 600 laser shots per pixel (50 laser shots per position of a random walk within each pixel). All ions in the *m/z* range 2000–25000 were detected with a sampling rate of 1 GHz. Additionally, ions below *m/*z 2000 were suppressed using the matrix suppression function of the Autoflex II, to limit detector-saturation-induced loss of sensitivity [34]. The experiments were externally calibrated using a MALDI preparation of a standard protein mixture adjacent to each tissue.

During definition of the imaging MS experiment the dataset is manually aligned with an optical image of the tissue, and were then subsequently aligned with an optical image of the H&E stained tissue (tissue stained after the imaging MS experiment [7]).

### Data preprocessing

Each pixel's spectrum was processed using a smoothing and baseline subtraction routine using FlexAnalysis. A Gaussian algorithm was used for mass spectral smoothing (width 2 *m/z*, 4 cycles) and a ConvexHullV3 algorithm was used for baseline subtraction. Mass spectral smoothing and background subtraction are now established preprocessing strategies [13], [14].

### Data reduction—feature identification and extraction

Data reduction was performed as previously described using custom scripts written in Matlab (v. 7.4.0. Mathworks) [35]. The algorithm is based on the calculation of multiple mass spectral representations of an imaging MS dataset, including representations that explicitly highlight localized features, followed by automated detection of the peaks present in each mass spectral representation. Peak detection used the LIMPC algorithm [36], a signal-to-noise threshold of 4 and a peak width of ±500 ppm. The peak-lists obtained from each mass spectral representation were then collated into a final dataset-specific peak-list, which was used to extract all features from the imaging MS dataset using an integration window of ±500 ppm.

For the simultaneous analysis of multiple imaging MS datasets the dataset-specific peak-lists were collated using a mass tolerance of 100 ppm into a final project-specific peak list, which was then used to extract all features from each imaging MS dataset (see Figure S1). The reduced imaging MS datasets were then merged into a single project dataset using pixel offsets. In this manner the multivariate techniques could be simultaneously applied to all imaging MS datasets in the project, thus enabling the MS profiles to be compared within and between each tissue's imaging MS dataset.

Note: data reduction via automated peak identification and extraction has the disadvantage that peaks below the S/N threshold, but which may contribute to the differentiation, are not included in the subsequent data analysis. While a lower S/N threshold may be used this can lead to a rapid increase in the amount of chemical noise retained in the dataset, which can undermine the ability of the statistical data analysis tools to differentiate between the different regions of the imaging MS datasets. As explained in the results section, the lower dataloads provided by data reduction are fundamental to the practical application of imaging MS-based molecular histology.

### Target images

**Figure 2:**
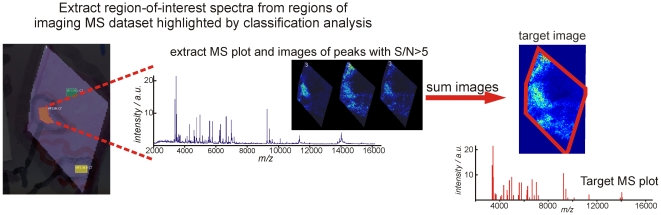
Creation of target images depicting the heterogeneity within imaging MS datasets of intermediate grade myxofibrosarcoma. A supervised classification analysis of the imaging MS datasets revealed intratumor heterogeneity. For each distinct region highlighted by the classification analysis region-of -interest mass spectra were extracted which contain all peptide and protein ions detected from that region of tissue. The images of all peaks with a S/N>5 were then extracted and summed together to form the target image (for testing the performance of the unsupervised data analysis routines). Y-axis labels, a.u. = arbitrary units.

To test the capabilities of a number of multivariate techniques to identify the heterogeneity in the imaging MS datasets target images were created based on our previous classification analysis of intermediate-grade myxofibrosarcoma [20]. A schematic of the creation of the target images is shown in Figure 2. The average mass spectrum was extracted from each region highlighted by the classification analysis. These region-of-interest spectra contain all of the peptide and protein ions that were detected in the regions highlighted by the classification analysis. The automated feature detection routine was then used to determine the principal peptide and protein ion peaks in each region-of-interest mass spectrum (signal-to-noise >5). The images of these principal contributors were then extracted from the imaging MS dataset and algebraically summed to form the target image. The associated MS plot, of the peaks containing a S/N>5, forms the target MS plot (Figure 2). In this manner the target images and MS plots contain the unrefined imaging MS heterogeneity detected by the classification analysis; unrefined because the target images include contributions from all peptide and protein ions, those responsible for the heterogeneity detected by the classification analysis and those with a more uniform distribution. These target images and the associated MS plots (containing the peaks with S/N>5), identified using a supervised analysis, were then used to gauge the ability of unsupervised methods to identify the intratumor heterogeneity.

### Statistical analysis algorithms

Six unsupervised data analysis algorithms were investigated for their ability to identify the endogenous molecular variation in the myxofibrosarcoma tissues. A brief summary plus references containing a detailed description of each algorithm are provided:-

- Principal Component Analysis: Performs linear orthogonal transformation of the data to maximize variance, resulting in a set of orthogonal principal components that describe the largest variance in the dataset (PC 1), the next largest variance (PC 2), and so on [37].
- Non-Negative Matrix Factorization: Decomposes the data into a sum of additive non-negative components (explicit requirement, scores and loadings must be non-negative) [38].
- Maximum Autocorrelation Factorization: Data is decomposed in similar manner to PCA, but the factorization is performed on a shift matrix, which is the data subtracted from a copy of itself shifted by one pixel [39].
- K-Means Clustering: Assigns each pixel to a predefined number of classes using the squared Euclidean distance between spectra [32].
- Fuzzy C-Means Clustering: Assigns each pixel to a predefined number of classes using the Euclidean distance between spectra, but individual pixels can occupy multiple classes [40].
- Probabilistic Latent Semantic Analysis: Statistical mixture model to divulge latent tissue-type specific molecular signatures [31]. Provides probability distributions that allow the peptides and proteins that discriminate specific tissue types to be determined.

All of the algorithms decompose the imaging MS datasets into a series of components (formally k-means and fuzzy c-means clustering demarcate the tissues into classes, for consistency we refer to them as components). Each data analysis method generates score images and loadings plots for each output component, referred to here as component images and component plots respectively. Component images are obtained by projecting each pixel's score onto its pixel coordinates. In imaging MS-based molecular histology regions displaying similar scores in the component images are considered to have correlated MS profiles. The component plot depicts an MS spectrum containing the MS features that contribute to the component.

### Statistical analysis implementation

The reduced data is extracted as a two dimensional matrix with the extracted peak intensities from each pixel arranged in rows and normalized to each pixel's total-ion-count. A second matrix contains the coordinates of each pixel. A mean-centering step is included as the first step of all analyses with the exception of non-negative matrix factorization and probabilistic latent semantic analysis as these techniques have the requirement of positive or zero values.

Principal component analysis was performed using the *princomp* routine from the Matlab statistics toolbox without modification. K-means clustering was performed using the *kmeans* routine, also from the Matlab statistics toolbox, using squared Euclidean distances. Non-Negative Matrix Factorization is based on David Ross's (University of Toronto: http://www.cs.toronto.edu/~dross/) implementation of Lee & Seung's Non-Negative Matrix Factorization algorithm [38]. The number of iterations was set to 100 and typically resulted in a stable solution. In cases where a convergence was not achieved the number of iterations was increased accordingly.

Probabilistic latent semantic analysis used the Matlab code of the Multidimensional Image Processing group at the University of Heidelberg [31]. The algorithm was used with default settings, a maximum number of iterations of 500 and the stopping criterion at a relative change of less than 10^−5^.

Maximum autocorrelation factor analysis was performed using the algorithm written by Allan Aasbjerg Nielsen of the Technical University of Denmark [41] modified for imaging MS data. All analyses were performed using the default shift matrix of one horizontal and one vertical shift with no additional scaling.

Fuzzy c-means was performed using the algorithm written by David Corney (University of Surrey, UK) [42] and the default degree of fuzziness, 1.25.

### Agreement plots

**Figure 3:**
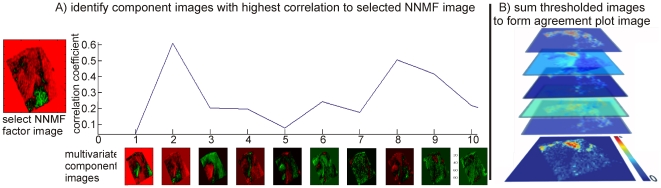
Automated selection of components displaying similar spatial features from multiple multivariate techniques. The component images of each multivariate method are used as a template to sort the components of the remaining data analysis algorithms. In this scheme the correlation between the NNMF components and those of each other algorithm are used to select the components with the highest similarity. The matching images are then thresholded and summed together to form the agreement plots.

The component images of each multivariate method, top 8 components, were used as a template to automatically select the matching images from the four other multivariate techniques. Figure 3A shows this selection using NNMF as the template. The component images were unfolded into one-dimensional vectors and the Pearson correlation between these vectors calculated. The component images from PCA, PLSA, fuzzy C-means, and MAF with the highest correlation (to a specific NNMF component image) were considered to be the best match. Note: the unfolded one-dimensional representations of the images were used for image correlation because of the irregularly shaped tissues typically analyzed in imaging MS experiments [13].

The matched component images with the highest correlation were then thresholded. Close examination of the image intensities indicated that the background signal was primarily lower than 40% of the maximum image intensity (see Supporting Information S1); pixels with an intensity >40% of the image's maximum intensity were assigned an intensity of one and all other pixels zero. These thresholded, matched component images were then summed together to form the agreement plots, Figure 3B.

To remove redundancy in the agreement plots (agreement plots showing identical spatial variation, due to iteratively using each multivariate method as an image template) a ‘cutdown’ routine was written that first sorts the agreement plots according to the number of correlated images, and then removes lower-ranked agreement plots that have a correlation coefficient greater than 0.7. MS outputs of the agreement analysis were obtained by averaging the loading plots from the matched outputs of the different multivariate methods (normalized to their basepeaks owing to the different scalings of their loadings plots).

## Results and Discussion

**Figure 4:**
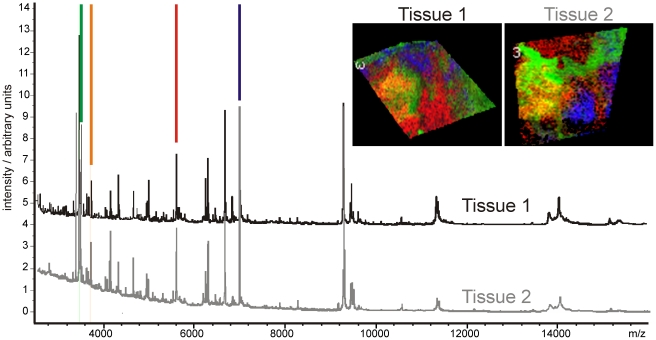
MALDI imaging MS datasets of intermediate grade myxofibrosarcoma.

Direct tissue analysis using MALDI-MS can generate MS profiles containing hundreds of peptide and protein ions. Imaging MS uses spatially resolved mass spectrometry to measure the distributions of these peptide and protein ions in tissue samples [4], [5]. Figure 4 shows two MALDI imaging MS datasets of intermediate-grade myxofibrosarcoma. 271 peaks (S/N>5) were detected in dataset #1 and 218 peaks were detected in dataset #2. Each MS peak corresponds to the detection of a different peptide/protein ion. The images display the distributions of four protein ions within these datasets and demonstrate the significant spatial heterogeneity that can be present in the peptide/protein ion distributions.

**Figure 5:**
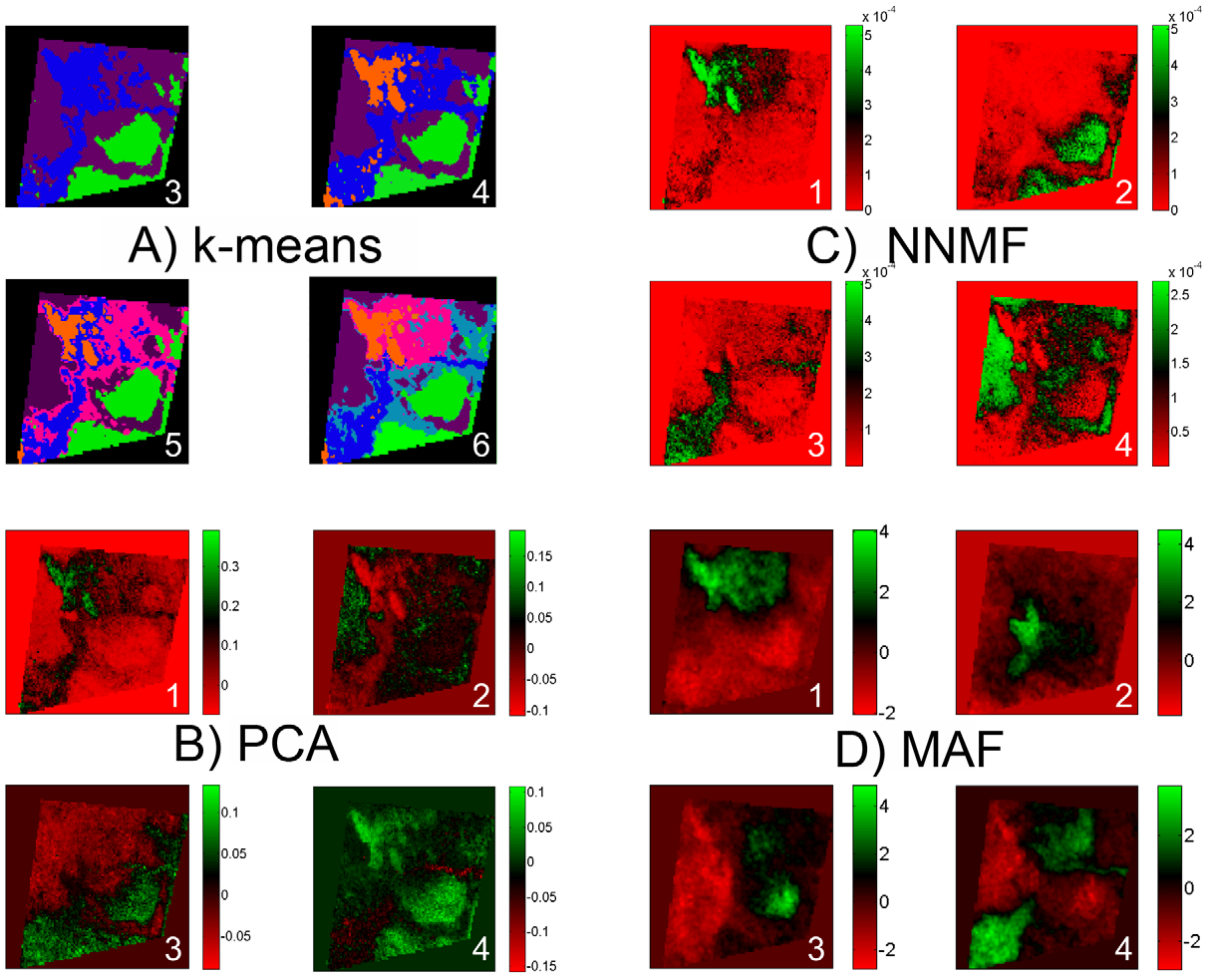
Imaging MS-based molecular histology can be dependent on the multivariate method. K-means clustering, principal component analysis, non-negative matrix factorization and maximum autocorrelation factor analysis of imaging MS datasets of intermediate grade myxofibrosarcoma. The apparent intratumor heterogeneity can be dependent on the multivariate method used for the analysis.

**Figure 6:**
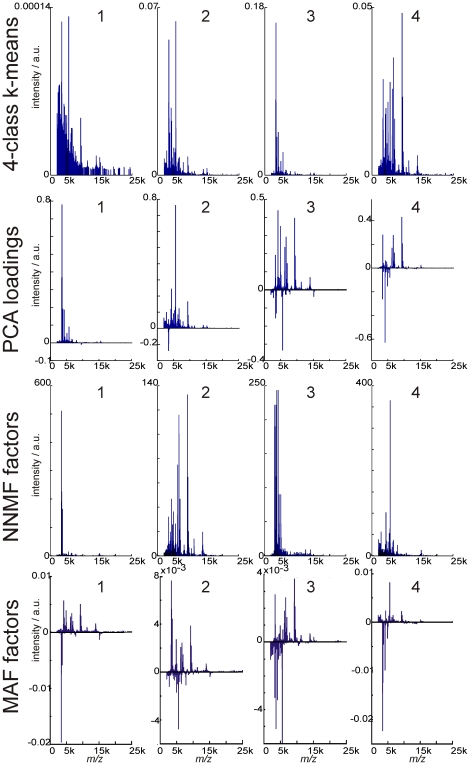
Component mass spectra from imaging MS-based molecular histology of intermediate grade myxofibrosarcoma using k-means clustering, principal component analysis, non-negative matrix factorization and maximum autocorrelation factorization. First row: cluster spectra following a 4-class k-means cluster analysis of imaging MS datasets of intermediate grade myxofibrosarcoma. Second row: Loading plots of first four principal components after principal component analysis. Third row: first four factors of non-negative matrix factorization. Final row: first four factors of maximum autocorrelation factorization. a.u. = arbitrary units.

The non-targeted nature of the imaging MS experiment means that previously unknown biomolecular variation may be uncovered. Imaging MS-based molecular histology consists of the application of statistical tools to identify regions of imaging MS datasets that exhibit distinct, correlated MS profiles. A variety of statistical tools have been investigated. Figure 5 shows the results of applying k-means clustering, principal component analysis (PCA), maximum autocorrelation factorization (MAF) and non-negative matrix factorization (NNMF) to an intermediate-grade myxofibrosarcoma imaging MS dataset. K-means clustering is a semi-supervised method that partitions the dataset into a predefined number of classes, but in which the apparent clustering is dependent on the number of classes. Figure 5A shows the resulting images for 3–6 classes (the file Supporting Information S1 includes the images for 2–10 classes). PCA, NNMF and MAF are unsupervised techniques that require no user input but which generate a series of component images containing correlations that are dependent on the multivariate technique as well as which component-output is investigated. Each of these data analysis techniques also provides a component plot mass spectrum that indicates which peptide and protein ion peaks were responsible for the observed correlations, Figure 6.

Figures 5 and 6 summarize the uncertainty raised in imaging MS-based molecular histology by the availability of multiple data analysis algorithms: the regions of the imaging MS dataset found to contain correlated biomolecular profiles, and consequently the peptide and protein ions that differentiate between these regions, can be dependent on the method chosen for the statistical analysis and which component output is selected. A description of the methodological differences between PCA, NNMF and MAF is included as supporting information (see Supporting Information S1).

Close inspection of Figure 5 reveals that the different multivariate techniques can highlight the same regions of the imaging MS dataset but, depending on the particular algorithm employed and which component image is selected, amalgamate them with additional regions. For example component image number 4 in PCA, number 2 in NNMF and number 3 in MAF all highlight nodules in the lower right corner of the imaging MS dataset. However there is little consistency regarding the association of these nodules with other regions of the imaging MS dataset.

**Figure 7:**
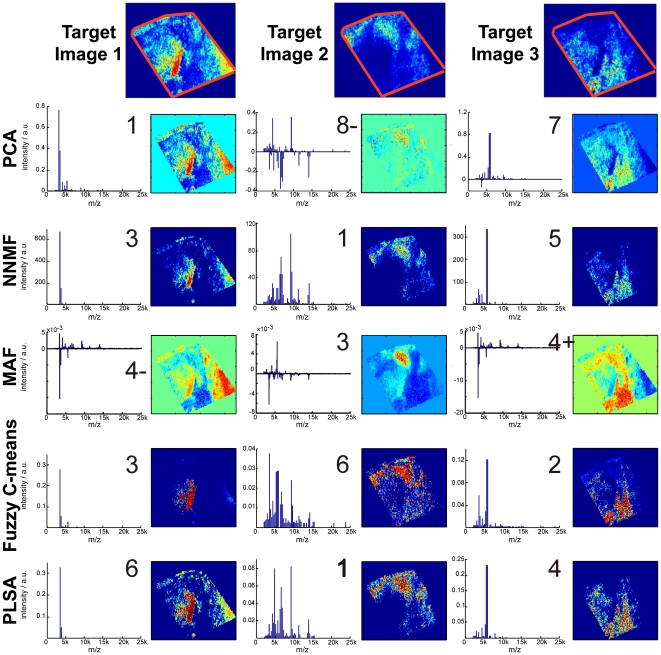
Identification of intratumor heterogeneity in imaging MS datasets by unsupervised multivariate analysis. Target images were created that contain the unrefined heterogeneity in an imaging MS dataset of intermediate grade myxofibrosarcoma. The outputs of principal component analysis, non-negative matrix factorization, maximum autocorrelation factor analysis, fuzzy c-means, and probabilistic latent semantic analysis were then examined to identify the components that contained the heterogeneity of the target images. The digit contained in the upper right corner of the component mass spectra indicates which *component* was used. Most data analysis techniques could reproduce the target images. When the component images reproduced the target images it can be seen that the component mass spectra contain the same peptide and protein ions. Note: PCA and MAF can have negative values, consequently the background surrounding the tissue (defined as zero intensity) can change color. Y-axis labels, a.u. = arbitrary units.

We hypothesized that the regions consistently identified as having distinct, correlated MS profiles by multiple multivariate techniques may provide a more accurate summary of the heterogeneity in the imaging MS dataset than any of the data analysis techniques used in isolation. To test the hypothesis a set of target images were created that depict the unrefined heterogeneity in an intermediate-grade myxofibrosarcoma dataset (see experimental). The component outputs of five multivariate techniques were then analyzed to identify which component images reproduced the target images. Figure 7 shows the target images and the corresponding component images and component mass spectra for PCA, NNMF, MAF, PLSA, and fuzzy c-means. Most of the multivariate techniques generated component images that contained the target images, the exception being PCA for target image 2. Where there is good agreement between the component images it can be seen that the corresponding component plot mass spectra also contain the same peptide and protein ions.

**Figure 8:**
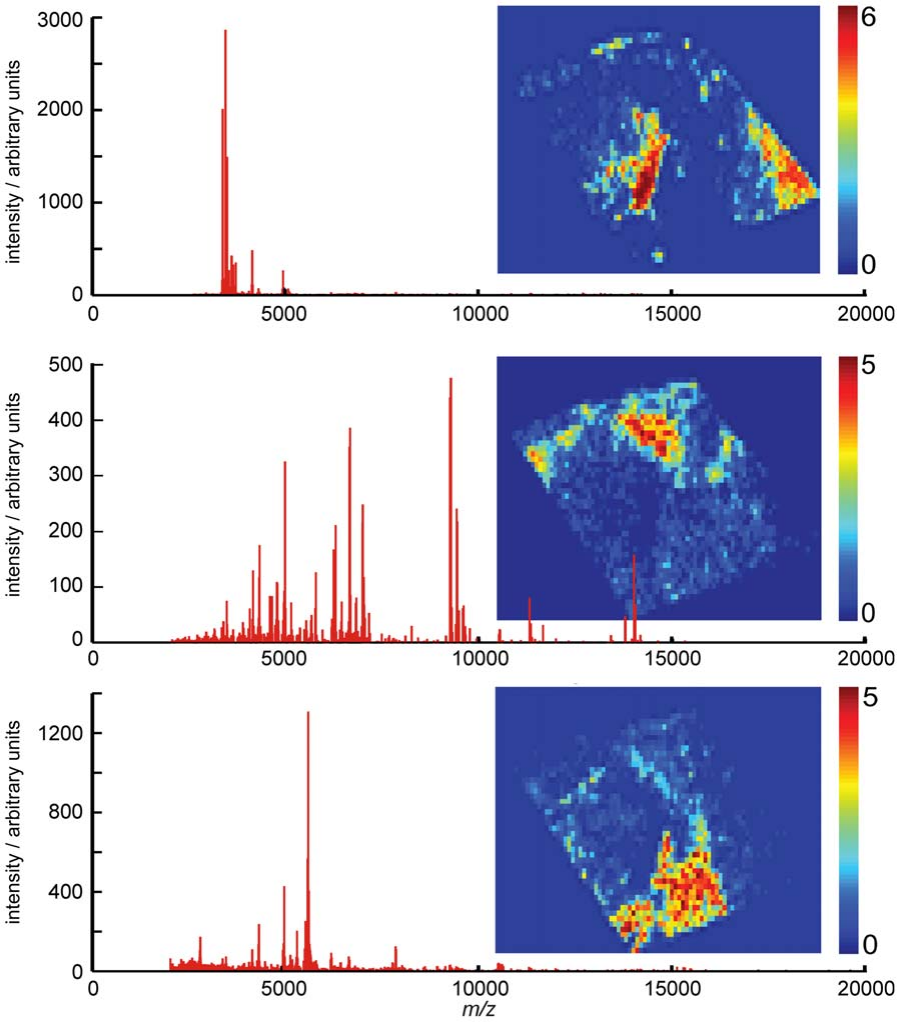
Agreement plots identify the distinct regions within imaging MS datasets. The agreement plot images and mass spectra show the regions of the imaging MS datasets consistently identified as unique by the different data analysis algorithms, and the peptide and protein ions consistently contributing to the differentiation. A comparison with Figure 7 clearly demonstrates that the agreement analysis images provide an accurate summary of the heterogeneity in the imaging MS dataset.

To highlight the regions of the imaging MS dataset corroborated by multiple data analysis techniques an image intensity threshold was applied to each component image containing the target image, and the thresholded images then summed together. An examination of the image intensities indicated that the background signal was typically lower than 40% of the maximum image intensity (see Supporting Information S1); accordingly pixels with an intensity >40% of the image's maximum intensity were assigned an intensity of one and all other pixels zero. Figure 8 shows such agreement images and their associated mass spectra for the three target images displayed in Figure 4. The regions of the imaging MS datasets corroborated by four or more data analysis techniques accurately summarize the target images. The correlation of the target images (and target spectra) with their matched component images and agreement plot images (and their associated spectra) are provided in table 1, as well as the mean correlation and standard deviation for each data analysis method. It can be seen that the agreement plots provide a consistently accurate depiction of the target images, and that the dispersion between the correlation coefficients is the lowest using the agreement analysis. It should be noted that the contrast of the agreement plot images is enhanced by the supposition of the results from the five multivariate methods; consequently the correlation with the agreement plot images will be slightly diminished.

**Table 1:** Correlation of target images and target mass spectra with their matching component images and component spectra from multivariate and agreement analysis.

	Target image 1		Target image 2		Target image 3		Mean	
	Image	MS	Image	MS	Image	MS	Image	MS
PCA	0.91	0.97	0.50	0.26	0.82	0.84	0.74	0.69
NNMF	0.94	0.94	0.90	0.63	0.85	0.79	0.89	0.79
MAF	0.69	0.94	0.58	0.65	0.59	0.80	0.62	0.79
Fuzzy c-means	0.72	0.97	0.72	0.54	0.87	0.72	0.77	0.74
PLSA	0.72	0.85	0.89	0.83	0.88	0.75	0.83	0.81
**Agreement**	**0.83**	**0.97**	**0.85**	**0.75**	**0.85**	**0.76**	**0.84**	**0.83**

For each target image (and associated mass spectrum) the Pearson correlation between the matching component images and mass spectra (loading plots) is provided. The latter two columns detail the mean correlation coefficient for each data analysis method.

**Note:** the limited dynamic range of an agreement plot based on threshold images and the wider dynamic range of the target image (discrete vs. continuous values) is not suited to a Pearson correlation calculation. Accordingly, the correlations have been calculated using an agreement plot based on non-thresholded data.

All of the above multivariate analyses were performed on reduced data obtained by automated feature detection and extraction. The significantly lower data load and data dimensionality, see table 2, enabled the multivariate methods to be applied on practical timescales. For example PCA, k-means clustering, MAF and NNMF could be applied to a single-tissue-dataset in just 0.3, 3.1, 6.7, and 18.3 s respectively using 64-bit Matlab running on a 64-bit Windows 7 workstation equipped with 64 Gb DDR3 1333 MHz RAM and one 2.66 GHz Xeon X5650 processor. The increased speed of the data analysis routines allowed the agreement analysis routine to be automated by using a correlation metric to identify which component images (of the different data analysis routines) identify similar regions of the imaging MS datasets, Figure 3. The entire agreement analysis workflow takes approximately 4 minutes per tissue. See the methods section for more details about data reduction and agreement analysis automation.

**Table 2:** Dataloads, number of variables and multivariate processing times of MALDI imaging MS datasets.

*Dataload per spectrum = 400 kB*					*Processor = 3.8 GHz Core i7*					
*Dataload per variable = 8 bytes*					*70 gflops max processing speed*					
	Tissue 1		Tissue 2		Tissue 3		Tissue 4		Total	
	*Raw*	*Red.*	*Raw*	*Red.*	*Raw*	*Red.*	*Raw*	*Red.*	*Raw*	*Red.*
**# pixels**	7363		9140		4479		8333		31156	
**# channels**	87220	254	87220	343	87220	271	87220	218	87220	358
**Dataload (MB)**	2876.2	14.3	3570.3	23.9	1749.6	9.3	3255.1	13.9	11451	61.3
**FLOP's** *	1.4e15	6.7e9	1.6e15	1.5e10	1.1e15	4.6e9	1.6e15	5.6e9	4.0e15	5.6e10
**Proc. time (s)**	20681	0.1	23385	0.2	16293	0.1	22157	0.1	56882	0.8
**Proc. time (days)**	0.2		0.3		0.2		0.3		0.7	

Summary of data processing parameters for imaging MS-based molecular histology of intermediate grade myxofibrosarcoma prior to feature extraction and following feature extraction.

*Number of floating point operations (FLOP's) given for a commonly used PCA algorithm, *flops = 14⋅k⋅N^2^+8⋅N^3^*, where *k* is the number of pixels and *N* the number of channels [33].

The multiplex multivariate agreement analysis routine has also been designed for the simultaneous analysis of multiple imaging MS datasets (for example from multiple patient tissue samples). Figure S1 illustrates how the automated feature detection and extraction algorithm is first used to acquire an aligned dataset that contains the distributions, in all datasets, of every peptide and protein ion detected in any of the datasets. These datasets are then merged into a single project-specific dataset using pixel offsets. In this manner the data analysis techniques could be simultaneously applied to all imaging MS datasets in a project (e.g. a small patient series), to examine the heterogeneity within and between the individual imaging MS datasets (from individual patient tissue samples). In its current form no preference is given for inter- or intra-dataset variation.

**Figure 9:**
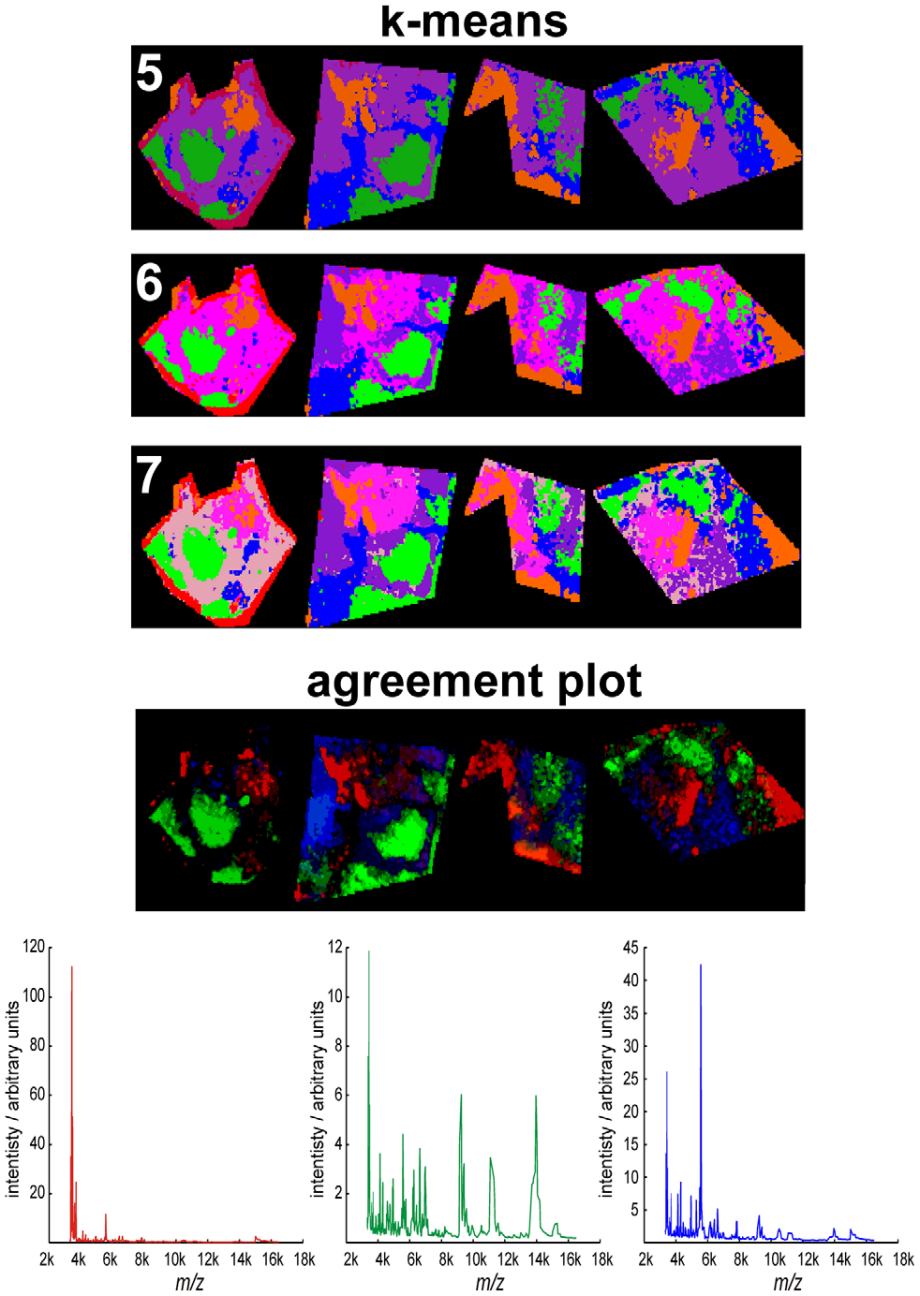
Intratumor heterogeneity identified in the imaging MS datasets of multiple intermediate grade myxofibrosarcoma patient tissue samples. Data reduction and integration of the imaging MS datasets from multiple patient tissue samples enables the data analysis routines to be used to simultaneously examine the heterogeneity within, and between, each patient's imaging MS dataset. Whereas the heterogeneity identified by k-means clustering is dependent on the user-defined number of classes, the agreement analysis reveals intratumor heterogeneity that is present in all datasets and which is corroborated by multiple data analysis techniques. A histological analysis revealed that the blue and red nodules are morphologically identical; however the imaging MS-based histology results clearly demonstrate they exhibit different MS profiles.

Imaging MS datasets of four patient tissue samples of intermediate-grade myxofibrosarcoma were merged into a single project-specific dataset and analyzed using the agreement analysis routine to highlight heterogeneity that was present in every patient's imaging MS dataset. Figure 9 shows a comparison of k-means clustering (5–7 classes) and the results of the agreement analysis (as an RGB false color image of three outputs of the agreement analysis). These agreement outputs have been selected to show nodules that are present in the imaging MS datasets of all four patient tissue samples. The file Supporting Information S1 includes eight outputs of the agreement analysis and includes nodules that are present in all datasets as well as those that are unique to datasets from individual patients.

The partitioning of the combined project-specific dataset by k-means clustering is dependent on the user-defined number of classes. As was observed in Figure 5 increasing the number of classes can highlight additional regions within the tissues. For the study of intratumor heterogeneity, especially across multiple patient tissue samples, it is difficult to know *a-priori* the number of classes that best describe the heterogeneity within the entire project-specific dataset. The agreement analysis, showing the concurrence between multiple data analysis techniques, has been specifically developed to highlight those nodules that are consistently identified as possessing distinct MS profiles. Figure 9 shows that the 6-class k-means clustering analysis also identified the regions highlighted by the agreement analysis, however without the agreement plots it would not be possible to affirm the number of classes.

The imaging MS datasets were aligned with optical images of the H&E stained tissues. A histological examination of the regions of tissue highlighted by the agreement analysis revealed that the green nodules were hypercellular with low amounts of collagenous matrix. In contrast the regions of tissue highlighted by the blue and red outputs of the agreement analysis were both characterized by low numbers of tumor cells and lots of hyaline collagen. The sharp boundaries between the hyper and hypocellular regions are characteristic of myxofibrosarcoma [29]. The regions of tissue highlighted by the blue and red outputs of the agreement analysis are not morphologically distinct, yet the results demonstrate that five independent multivariate techniques concur that their MS profiles are distinct. The agreement plot mass spectra, also included in Figure 9, clearly show the different MS profiles of the regions highlighted by the agreement analysis.

To date, imaging MS-based molecular histology analyses have concerned tissue samples with well differentiated morphologies (e.g. mouse brain [32], differentiating necrotic from viable tumor [31]), enabling the results to be compared with the tissue's morphology, in part because of the uncertainty raised by the availability of multiple data analysis algorithms. The agreement analysis reported here begins to address this uncertainty by explicitly highlighting those regions of the imaging MS datasets identified as unique by multiple data analysis algorithms, the results demonstrate that this provides an accurate summary of the dataset's heterogeneity. This corroboration enables imaging MS-based histology analysis of tissues that are not histologically distinct (and thus require a different form of corroboration).

The intratumor heterogeneity revealed in the imaging MS datasets of intermediate grade myxofibrosarcoma provides further evidence that imaging MS-based molecular histology may complement current histopathological practice by revealing underlying molecular changes that have not been observed using established histological and histochemical methods.

**Figure 10:**
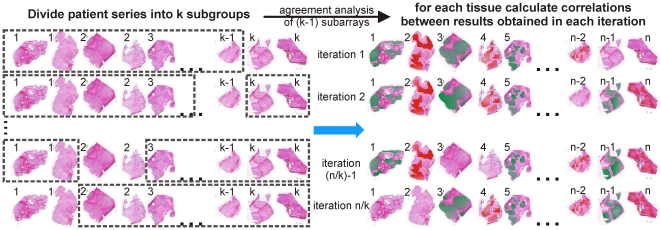
Cross-validated imaging MS-based molecular histology. k-fold cross-validation of imaging MS-based molecular histology. The imaging MS datasets from a large patient series are randomly split into k sub-groups and the agreement analysis iteratively performed on datasets containing (k-1) subgroups. The regions identified with each iteration can then be compared by calculating their correlation.

The agreement analysis routine provides the capability to summarize the heterogeneity within and between the imaging MS datasets of multiple tissue samples. Each of the regions identified by imaging MS-based molecular histology analysis contains many hundreds of pixels per tissue, and consequently are also characterized by many hundreds of individual MS measurements per tissue. The next step in the development of imaging MS-based molecular histology as a complementary histological technique will be to validate the findings using a large patient series, and to ascertain the origin of the heterogeneity detected by imaging MS (recall that through ionization biases imaging MS results are affected by the underlying chemical composition of the tissue, even though many of the chemical species are not represented in the mass spectrum). When used to differentiate between morphologically overlapping/identical tissues it will not be possible to refer to a histological analysis to determine performance metrics, as is used in imaging MS-based biomarker discovery experiments [8]. A k-fold cross-validation strategy [43] would ensure the results of the imaging MS-based molecular histology analysis are not dependent on which tissue's are contained in the patient series, Figure 10. This could then followed by a laser-capture microdissection, quantitative LC-MS analysis of the cross-validated regions, to provide independent confirmation of the observed heterogeneity as well as a more in-depth analysis of their proteome/metabolome/lipidome to ascertain its origin.

### Conclusion

Imaging MS-based molecular histology consists of the application of statistical tools to identify regions of imaging MS datasets that exhibit distinct, correlated MS profiles. When aligned with optical images of the tissue this enables the tissues to be annotated solely on the basis of these correlated profiles. Here it is demonstrated that the agreement of multiple data analysis algorithms provides an accurate summary of the spatio-chemical variation within in the dataset. When applied to imaging MS datasets of intermediate-grade myxofibrosarcoma distinct nodules were revealed in histologically identical tumor tissue, and confirmed in multiple patient tissue samples. These results highlight the potential of imaging MS-based molecular histology to complement established histological and histochemical methods, and begin to address some of the requirements for its wider implementation. To aid its further development Supporting Information S2 contains detailed instructions and Supporting Information S3.tar contains the Matlab code and an example reduced dataset.

## Supporting Information

Figure S1 **Reduction and integration of multiple imaging MS datasets.** An automated feature detection routine, based on the calculation of four different mass spectral representations for improved feature detection, is applied to each imaging MS dataset. The resulting experiment specific peaks lists are then collated into a project-specific peak list, which is used to extract the images of every feature, detected in any dataset, from all datasets. A set of pixel offsets are then used to integrate the reduced datasets into a combined, project specific dataset. Y-axis labels, a.u. = arbitrary units.
(TIF)

Supporting Information S1 A detailed outline of the ImagePrep settings used for matrix deposition.
(DOC)

Supporting Information S2 Detailed instructions.
(DOC)

Supporting Information S3 Matlab code and an example reduced dataset.
(TAR)
