# Supplementary figures and images for: Multiple Statistical Analysis Techniques Corroborate Intratumor Heterogeneity in Imaging Mass Spectrometry Datasets of Myxofibrosarcoma
Source: PLoS One. 2011 Sep 29;6(9):e24913. doi: 10.1371/journal.pone.0024913 (PMC3183001; doi:10.1371/journal.pone.0024913)

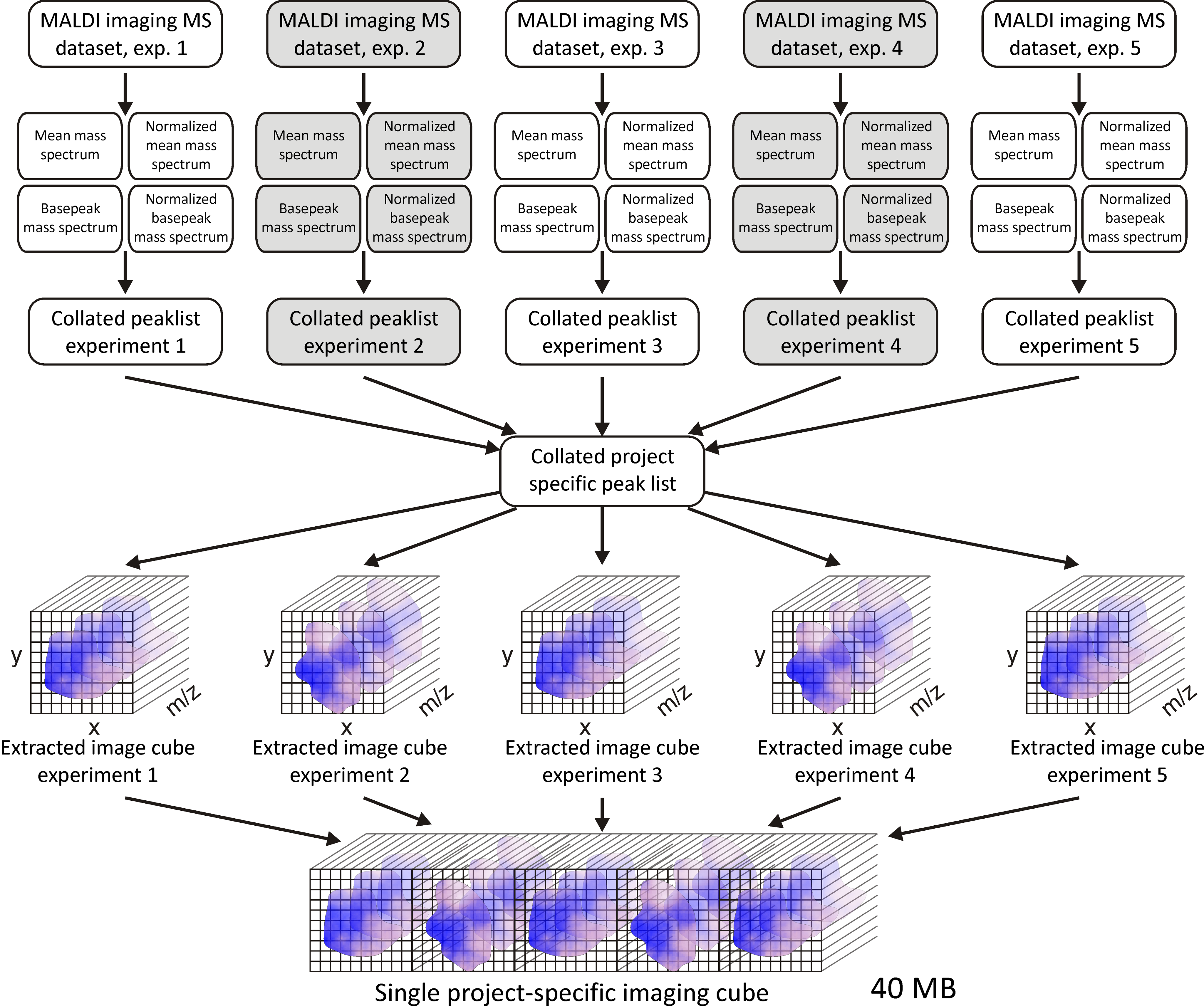

Supplement: Figure S1 — Reduction and integration of multiple imaging MS datasets. An automated feature detection routine, based on the calculation of four different mass spectral representations for improved feature detection, is applied to each imaging MS dataset. The resulting experiment specific peaks lists are then collated into a project-specific peak list, which is used to extract the images of every feature, detected in any dataset, from all datasets. A set of pixel offsets are then used to integrate the reduced datasets into a combined, project specific dataset. Y-axis labels, a.u. = arbitrary units. (TIF) [file pone.0024913.s001.tif]
