# Supplementary material for: Multiple Statistical Analysis Techniques Corroborate Intratumor Heterogeneity in Imaging Mass Spectrometry Datasets of Myxofibrosarcoma
Source: PLoS One. 2011 Sep 29;6(9):e24913. doi: 10.1371/journal.pone.0024913 (PMC3183001; doi:10.1371/journal.pone.0024913)
Supplement: Supporting Information S1 — A detailed outline of the ImagePrep settings used for matrix deposition. (DOC) [file pone.0024913.s002.doc]

**Multiple Statistical Analysis Techniques Corroborate Intratumor Heterogeneity in Imaging Mass Spectrometry Datasets of Myxofibrosarcoma**

Supplementary Data:

|  | Thickness | Nebulization | Incubation | Dry |
| --- | --- | --- | --- | --- |
| Phase 1 | Matrix layer thickness 0.1V  2-3 cycles | 20% power, 10% modulation  1.5s spray | 10s | 30s dry |
| Phase 2 | Matrix layer thickness 0.3V  2-8 cycles | 20% power, 10% modulation  2s spray | 15s | 45s dry |
| Phase 3 |  | -- | -- | 60s |
| Phase 4 | Matrix layer thickness 0.25V  2-12 cycles | 17% power, 10% modulation  Sensor 0.15V drop per cycle | 10s | Complete dry every cycle |
| Phase 5 | Matrix layer thickness 0.2V  12-32 cycles | 20% power, 10% modulation  Sensor 0.1V drop per cycle | 20s | 30% dry; complete dry every 2^nd^ cycle |
| Phase 6 | Matrix layer thickness 0.25V  4-15 cycles | 20% power, 10% modulation  Sensor 0.2V drop per cycle | 20s | 40% dry; complete dry every 3^rd^ cycle |
| Phase 7 | Matrix layer thickness 0.25V  4-15 cycles | 20% power, 10% modulation  Sensor 0.2V drop per cycle | 20s | 40% dry; complete dry every 5^th^ cycle |

.

**Table 1.** ImagePrep method for matrix deposition.

**2**

**10**

**9**

**8**

**5**

**4**

**3**

**6**

**7**


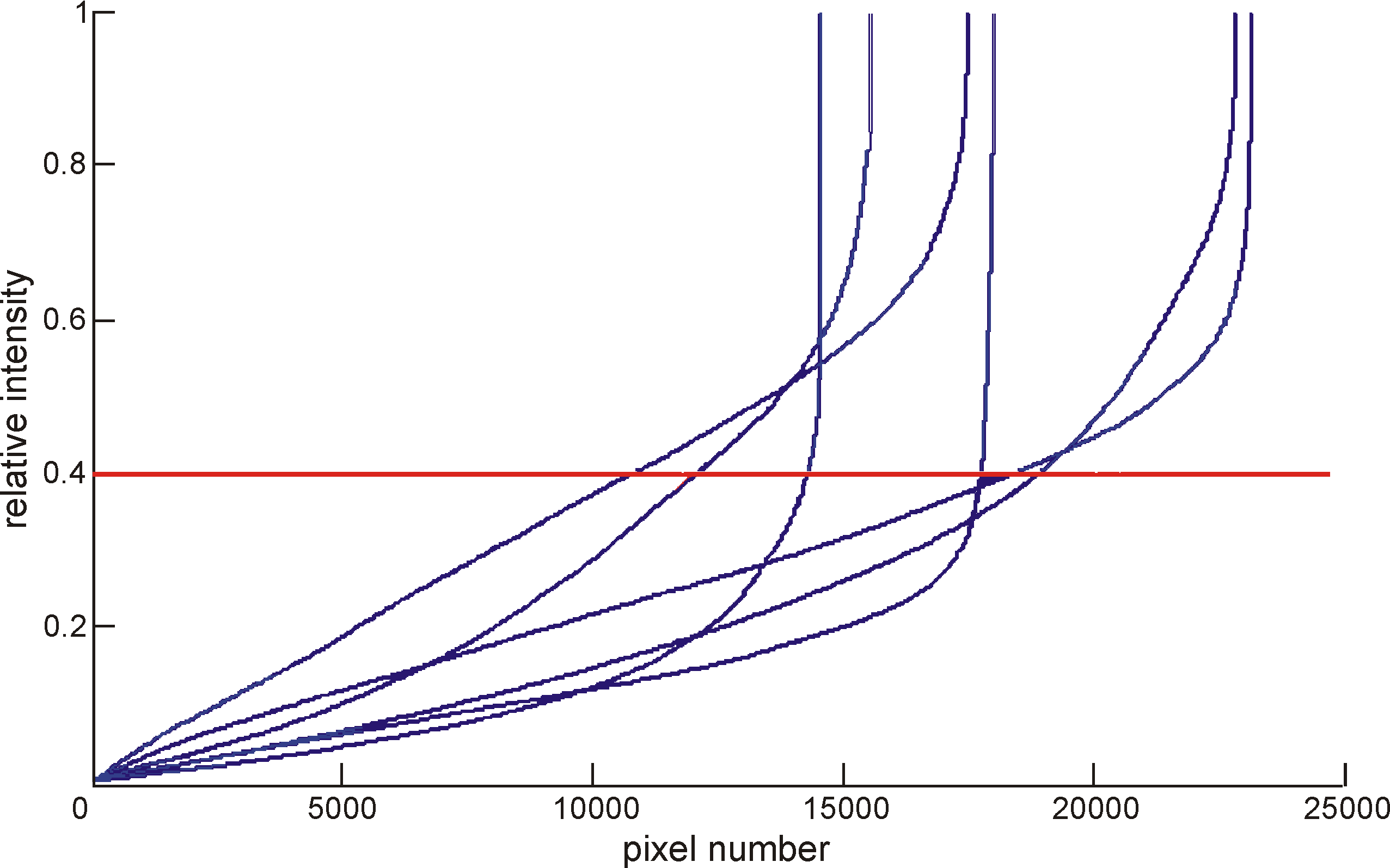


**Supplementary Figure 1.** Line graphs of pixel relative intensities versus pixel number of component images (ordered according to increasing intensity). The graphs indicate that the background signal is mostly less than 40%, and so 40% was used as the image intensity threshold for the agreement analysis.

**Methodological description of figure 2.**

Figure 2 of the manuscript shows the results of K-means clustering, principal component analysis, maximum autocorrelation factor analysis, and non-negative matrix factorization of the intermediate-grade myxofibrosarcoma tissue sample. K-means clustering partitions the tissue into a predetermined number of classes based on the similarity (Euclidean distance) of each pixel’s peptide and protein profile.^1^ When applied to the intermediate-grade myxofibrosarcoma tissue biomolecularly distinct nodules within the tumor are revealed but the number of discrete nodules is dependent on the user-defined number of classes. Principal component analysis (PCA) maximizes the variance in the dataset by calculating linear combination of the original variables (*i.e.* m/z values) to create new variables, the principal components (PC’s).^2^ When applied to an imaging MS dataset PCA scores each pixel according to its value in the transformed variable space and so generates a scores-plot image for each output component. Figure 2B shows the scores-plot images for PC’s 1 to 4. Whereas k-means clustering demarcates the entire image into a single image (depicting each pixel’s group membership) PCA generates an image for each component output, thus raising the question if regions associated in PC3 but not PC4 are truly biomolecularly correlated, Figure 2B.

PCA generates pixels with ‘negative’ as well as ‘positive’ scores. The score has no direct physical meaning; it is the pixel’s value on the new coordinate system (the PC). The multivariate technique non-negative matrix factorization (NNMF) explicitly constrains the factors to non-zero values (both pixel scores and contribution of each variable, *m/z*, to the ‘components’), and thus provides images and spectra that are more readily interpreted than PCA. Figure 2C shows components 1 to 4 of an NNMF analysis of the same intermediate grade dataset.

K-means clustering, PCA and NNMF treat each pixel’s mass spectrum as independent measurements and so do not take into account any spatial relationships, for example between neighboring pixels. The final multivariate technique in Figure 2, maximum autocorrelation factor (MAF) analysis explicitly incorporates this spatial aspect.^3^ The basic assumption is that real signals exhibit high autocorrelation (between adjacent pixels), whereas noise exhibits low autocorrelation. The first MAF component is the linear combination of original variables that contains the maximum autocorrelation between neighboring pixels. Subsequent components are the linear combinations of the original variables that contain maximum autocorrelation subject to the constraint that they are orthogonal to the previous MAFs. In imaging MS maximization of autocorrelation between adjacent pixels should highlight regions of tissue that exhibit similar peptide and protein profiles.

Figure 4 of the manuscript also includes the data analysis techniques fuzzy c-means and probabilistic latent semantic analysis. Fuzzy c-means clustering partitions the dataset into a number of classes defined by Euclidean distances. However each pixel can occupy multiple classes enabling the underlying molecular patterns to be identified^4^ rather than forcing each pixel into a single specific class (k-means clustering). Probabilistic latent semantic analysis (PLSA) is based on a mixture decomposition of latent classes, and maps each latent class throughout the tissue.^5^ The principal advantage of PLSA is that it provides a probability distribution in the spectral dimension, enabling a statistically more rigorous interpretation of the class spectra. It has been shown to be equivalent to NNMF using the Kullback-Leibler divergence as the cost function.^6^


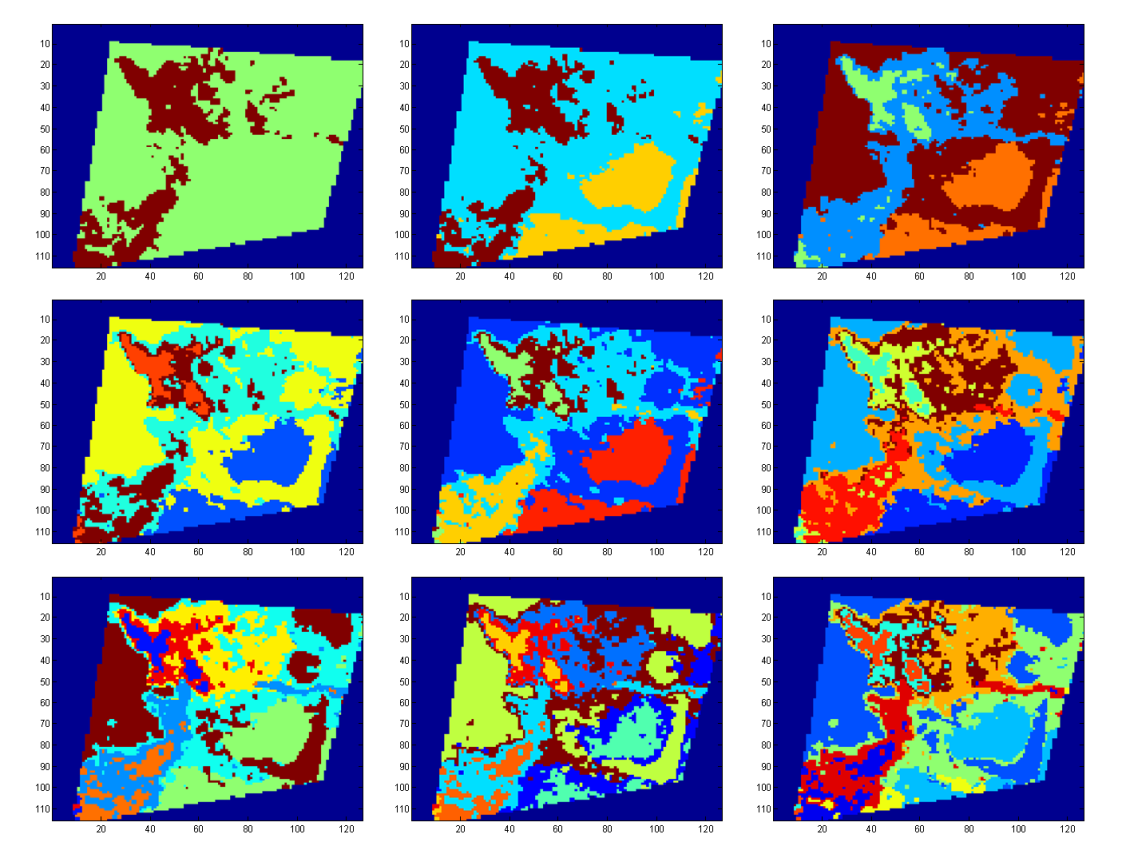


**2**

**10**

**9**

**8**

**5**

**4**

**3**

**6**

**7**

**Supplementary Figure 2.** K-means clustering of an intermediate grade myxofibrosarcoma tissue. The number and location of biomolecularly distinct regions is dependent on the number of user-defined classes. Insert is number of classes.


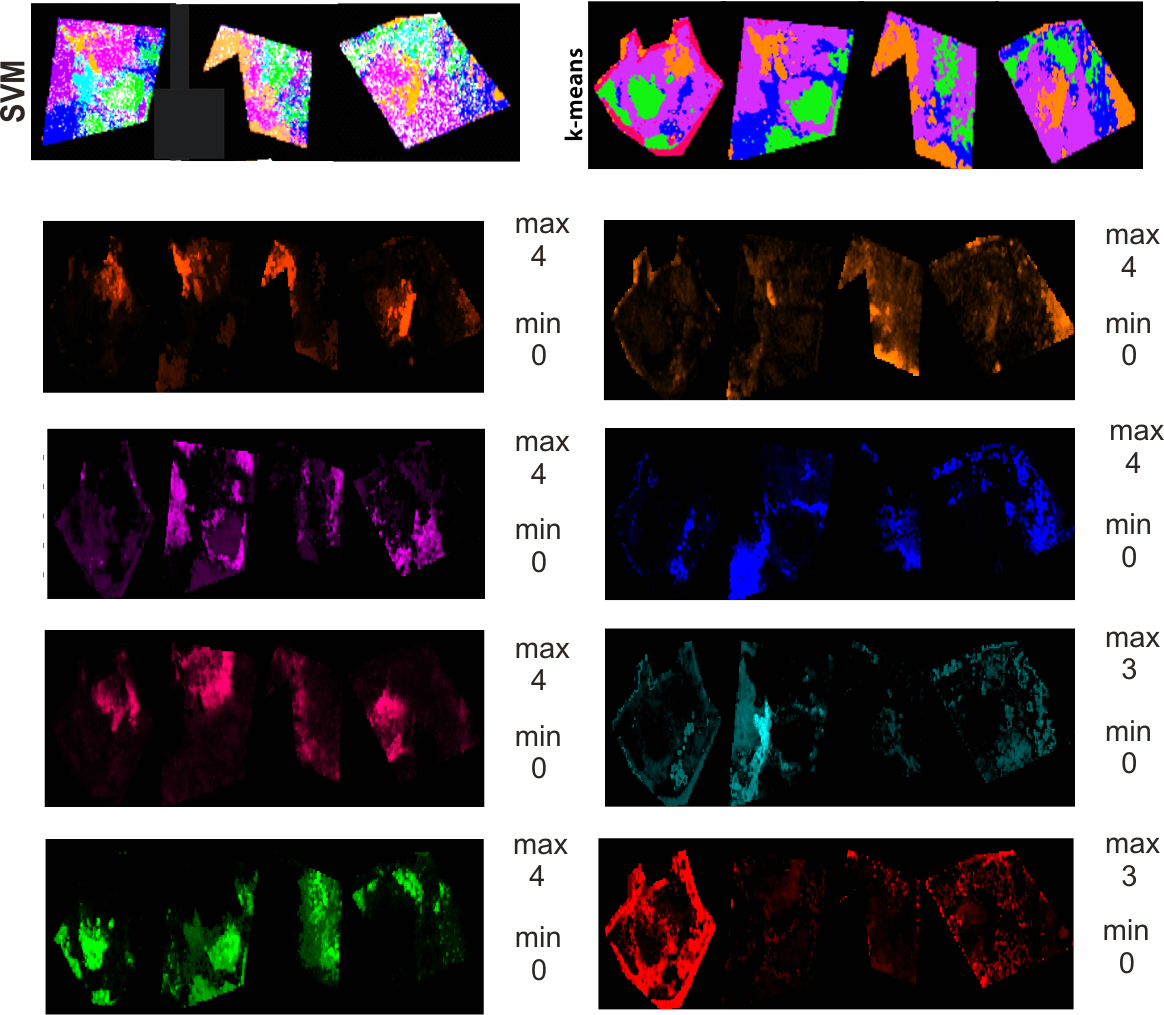


**Supplementary Figure 3.** Eight outputs of the multiplex multivariate agreement analysis applied to the unified dataset of all patient samples identifies biomolecularly distinct nodules that are present in all patient samples, as well as regions of tissues that are unique to specific patients. This is crucial in order to differentiate nodules that may be associated with tumor development from individual variation.

1. Alexandrov, T.; Becker, M.; Deininger, S.-O.; Grasmair, G.; von Eggeling, F.; Thiele, H.; Maass, P., Spatial Segmentation of Imaging Mass Spectrometry with Edge Preserving Image Denoising and Clustering. *J. Proteome Res.* **2010,** 9, 6535-6546.

2. Broersen, A.; van Liere, R.; Altelaar, A. F. M.; Heeren, R. M. A.; McDonnell, L. A., Automated, Feature-Based Image Alignment for High-Resolution Imaging Mass Spectrometry of Large Biological Samples. *J. Am. Soc. Mass Spectrom.* **2008,** 19, (6), 823-832.

3. Switzer, P., Min/Max Autocorrelation Factors for Multivariate Spatial Imagery. In *Computer Science and Statistics*, Billard, L., Ed. Elsevier: Amsterdam, 1985; pp 13-16.

4. Dunn, J. C., A Fuzzy Relative of the ISODATA Process and Its Use in Detecting Compact Well-Separated Clusters. *J. Cybernetics* **1973,** 3, 32-57.

5. Hanselmann, M.; Kirchner, M.; Renard, B. Y.; Amstalden, E. R.; Glunde, K.; Heeren, R. M. A.; Hamprecht, F. A., Concise Representation of Mass Spectrometry Images by Probabilistic Latent Semantic Analysis. *Anal. Chem.* **2008,** 80, (24), 9649-9658.

6. Gaussier, E.; Goutte, C. In *Relation Between PLSA and NMF and Implications*, SIGIR '05 Proceedings of the 28th Annual International ACM SIGIR Conference on Research and Development in Information Retrieval, Salvador, Brazil, 2005; Salvador, Brazil, 2005.
