# Supplementary material for: Multiple Statistical Analysis Techniques Corroborate Intratumor Heterogeneity in Imaging Mass Spectrometry Datasets of Myxofibrosarcoma
Source: PLoS One. 2011 Sep 29;6(9):e24913. doi: 10.1371/journal.pone.0024913 (PMC3183001; doi:10.1371/journal.pone.0024913)

**Multiplex multivariate analysis instructions.**


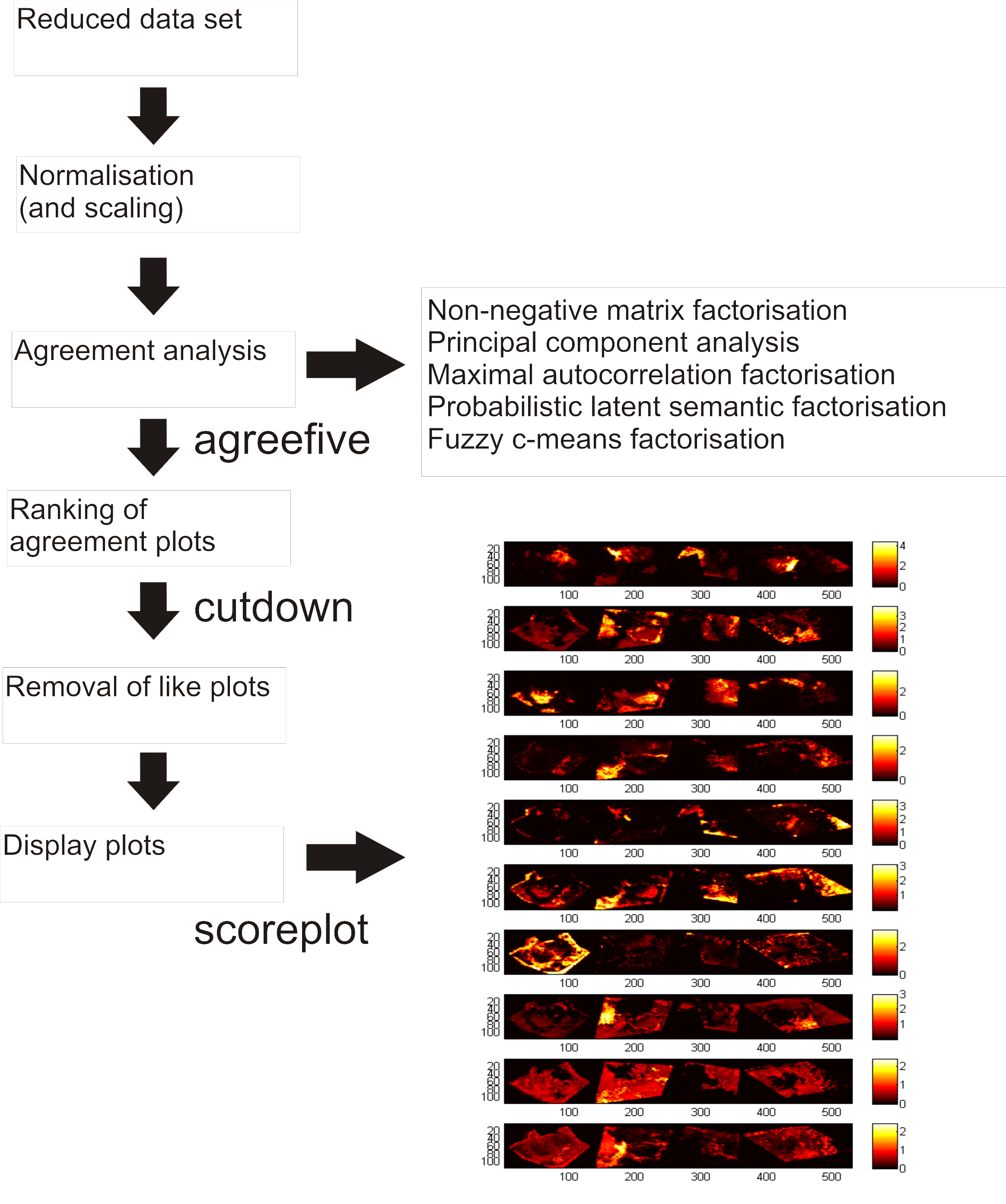


**Instructions Figure 1.** Schematic of data analysis workflow of multiplex multivariate agreement analysis.

Prior to multiplex multivariate agreement analysis the image data from each tissue was extracted using an automated feature identification and feature extraction routine (McDonnell *et al.* J. Am. Soc. Mass Spectrom. **2010**, *21*, 1969-1978). The principal outputs of this routine are:-

1. An *M*×*N* matrix, where *M* is the number of peaks detected in the imaging MS dataset and *N* is the number of pixels.
2. A 2×N matrix containing the coordinates of each pixel.
3. A 3×N matrix containing the *m/z* of the peaks detected in the imaging MS dataset, and the upper and lower bounds used for peak extraction (normally defined with a user defined peak width in *ppm*).
4. A single digit indicating the tissue number (for multi-tissue analysis).
5. The imaging MS experiment filename and data location.
6. A vector containing the total-ion-count of every pixel.

For multi-tissue analysis the peak lists from each tissue are collated and then used to extract the all peaks (detected in any tissue) from all of the tissue samples.

The data from all tissues (now with identical peak lists) are concatenated into a single data cube, while retaining the pixel locations and tissue number of every pixel. This data can then be (TIC or otherwise) normalised and scaled if required.

The agreement plot routine (*agreement.m***)** runs five multivariate analysis techniques, correlates the results from all methods and produces the agreement plots

**The five multivariate routines are:**

Principal component analysis Matlab princomp

Non-negative matrix factorisation David Ross’s (University of Toronto: http://www.cs.toronto.edu/~dross/) implementation of Lee & Seung's Non-Negative Matrix Factorization algorithm^26^

Fuzzy c-means factorisation Algorithm written by David Corney (University of Surrey, UK) ^28^ and the default degree of fuzziness, 1.25.

Maximal autocorrelation factorisation algorithm written by Allan Aasbjerg Nielsen of the Technical University of Denmark ^27^ modified for MALDI MS data (modified to accept raw data)

PLSA Matlab code of the Multidimensional Image Processing group at the University of Heidelberg ^17^

All MVA analysis conditions are run with set values ( these can be changed within the ‘agreefive’ Matlab m.file). For PCA and MAF, which generate positive and negative scores images, the positive and negative images are treated independently. Consequently, seven images are generated for each component output.

The next stage is iterative correlation of all component outputs, using ‘agreementP’

Each component image from each MVA technique is given a correlation score against all other output images, the closest match from each technique is then thresholded to remove noise (<40%) then added together to form an agreement plot for that output.

In order to present the most relevant data the ‘cutdown’ routine organises all the agreement plots by their top score and through another correlation routine sequentially compares the agreement plots from 1:n removing lower ranked scores that match within 70%. [This is variable within the script].


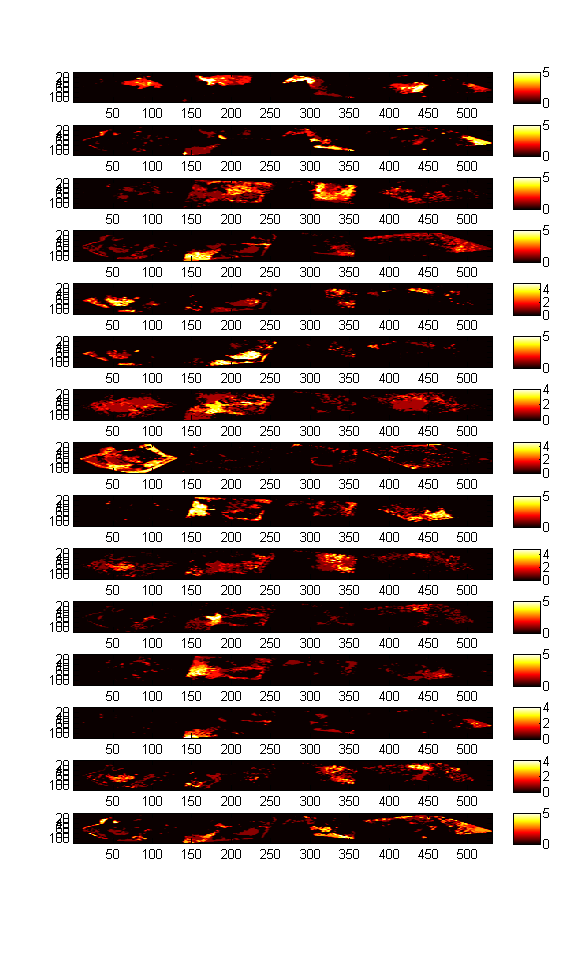


The final routine, scoreplot is a simple viewing script that allows all of the chosen agreement plots to be visualised together.

*

A test (reduced) data file is provided.

Test data: tissue 200509

Raw image data folder size 6.1GB

Reduced data size on disk 9.1MB

Agreement plot run with 6 components

Agreement plot running time 77.1 seconds

Cut down to 7 agreement plots

Output from automated method


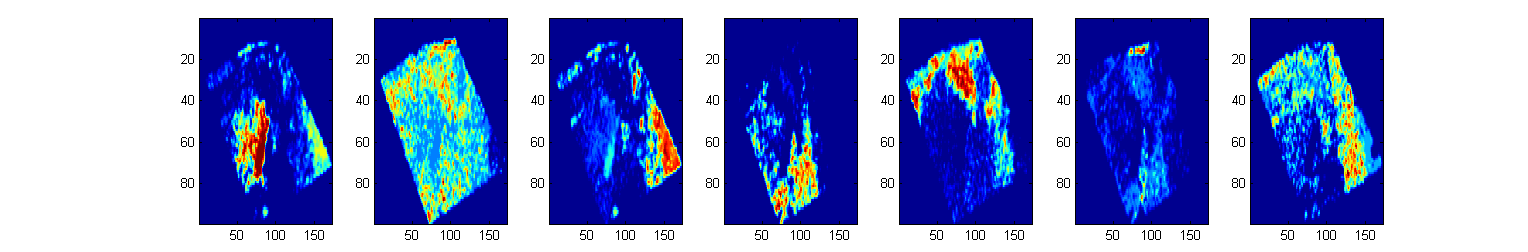

Supplement: Supporting Information S2 — Detailed instructions. (DOC) [file pone.0024913.s003.doc]
